# High burden of diabetes and prediabetes among cancer patients at University of Gondar comprehensive specialized hospital, Northwest Ethiopia

**DOI:** 10.1038/s41598-023-36472-y

**Published:** 2023-06-09

**Authors:** Haileab Fekadu Wolde, Meseret Derbew Molla, Hailu Aragie, Dagnew Getnet Adugna, Ephrem Tafesse Teferi, Endalkachew Belayneh Melese, Yohannes Awoke Assefa, Habtu kifle, Yilkal Belete Worku, Daniel Gashaneh Belay, Anteneh Ayelign Kibret

**Affiliations:** 1 Department of Epidemiology and Biostatistics, Institute of Public Health, College of Medicine and Health Sciences, University of Gondar, Gondar, Ethiopia; 2 Department of Human Anatomy, School of Medicine, College of Medicine and Health Sciences, University of Gondar, Gondar, Ethiopia; 3 Department of Biochemistry, School of Medicine, College of Medicine and Health Sciences, University of Gondar, Gondar, Ethiopia; 4 Department of Internal Medicine School of Medicine, College of Medicine and Health Sciences, University of Gondar, Gondar, Ethiopia; 5 Department of Occupational Therapy School of Medicine, College of Medicine and Health Sciences, University of Gondar, Gondar, Ethiopia

**Keywords:** Oncology, Cancer

## Abstract

Cancer and diabetes mellitus (DM) are diagnosed within the same individual more frequently and share common risk factors. Although diabetes among cancer patients may result in more aggressive clinical courses of cancer, there is limited evidence about its burden and associated factors. Hence, this study aimed to assess the burden of diabetes and prediabetes among cancer patients and its associated factors. Institution-based cross-sectional study was conducted at the University of Gondar comprehensive specialized hospital from 10 January to 10 March 2021. A systematic random sampling technique was used to select 423 cancer patients. The data was collected using a structured interviewer-administered questionnaire. Prediabetes and diabetes diagnosis was made based on World Health Organization (WHO) criteria. Bi-variable and multivariable binary logistic regression models were fitted to identify factors associated with the outcome. Adjusted Odds Ratio (AOR) with a 95% confidence interval was estimated to show the direction and strength of associations. Variables with a p-value less than 0.05 in the multivariable model were considered significantly associated with the outcome. The final analysis was based on 384 patients with cancer. The proportion of prediabetes and diabetes was 56.8% (95% CI 51.7, 61.7) and 16.7% (95% CI 13.3, 20.8), respectively. Alcohol consumption was found to increase the odds of elevated blood sugar among cancer patients (AOR: 1.96; 95%CI: 1.11, 3.46). The burden of prediabetes and diabetes is alarmingly high among cancer patients. Besides, alcohol consumption was found to increase the odds of having elevated blood sugar among cancer patients. Hence, it is essential to recognize cancer patients are at high risk of having elevated blood sugar and design strategies to integrate diabetes and cancer care.

## Introduction

Noncommunicable diseases (NCDs) are the leading causes of death globally, killing more people each year than all other causes combined^1^. The combined burden of these diseases is rising fastest among lower-income countries, including Ethiopia^2^. Of these diseases, cancer and Diabetes Mellitus (DM) are challenging the health system in Ethiopia. Although there is limited data, the Federal Ministry of Health (FMOH) estimated that there could be more than 150,000 cancer cases in Ethiopia each year^3^. Besides, of total national mortality in the country, 5.8% is caused by cancer^4^. On the other hand, it is estimated that developing countries, including Ethiopia, bear 77% of the global burden of the DM epidemic in the twenty-first century^5^.

Cancer and DM are diagnosed within the same individual more frequently than expected by chance, suggesting a common underlying mechanism^6^. About 26.9% of all people over 65 have diabetes and 60% have cancer worldwide. Overall, 8 to18% of cancer patients have DM^7^.

Diabetes and cancer share common risk factors such as increased age, obesity, physical inactivity, poor diet, alcohol, and smoking^6^. Although most evidence shows DM to be a risk factor for different types of cancer^8–10^, some findings suggest that the relationship is bidirectional^11^. Unfortunately, many cancer-fighting treatments are linked to diabetes and the incidence of diabetes and prediabetes is significantly higher among people with cancer compared with those who don’t have cancer^12–14^.

Some epidemiological studies suggest that diabetes significantly increases mortality in patients with cancer^8,15–17^ and is also the common cause of non-cancer mortality^18^. Diabetes may result in a more aggressive clinical course of cancer, strengthening its metastatic potential and favoring cancer growth by making the host organism less resistant to cancer progression, possibly by known impaired immune function in diabetes^7,17,19^. It is also associated with physiologic distress and decreased quality of life^20^ among cancer patients. On the other hand, DM significantly negatively impacts cancer patients' treatment outcomes^21^.

NCDs, including cancer and DM, are among the health targets of the Sustainable Development Goals^22^. Despite the devastating consequence of diabetes among cancer patients, there is limited evidence about its burden and associated factors. Besides, available evidence focuses on specific types of cancer. Hence, this study aimed to assess the burden of diabetes and prediabetes among cancer patients and its associated factors.

## Methods

### Study design and setting

An institution-based cross-sectional study was conducted from 10 January to 10 March 2021 among cancer patients on treatment at the University of Gondar Comprehensive Specialized Hospital (UoGCSH). The hospital was established in 1954 and is in the Central Gondar administrative zone, Amhara National Regional State, about 750 km Northwest of Addis Ababa, the capital city of Ethiopia. According to the 2015 population projection of major cities in Ethiopia, the total population of Gondar town was estimated to be 323,900. Currently, Gondar town has one Referral Hospital and eight government Health Centers. The University of Gondar Referral Hospital is a teaching Hospital that serves more than five million people in the North Gondar zone and people of the neighboring zones. The hospital has one Oncology ward. The ward serves more than 1000 cancer patients per year. The hospital's oncology unit currently has ten beds for managing cancer patients.

### Population and sample

The source population for this study is all cancer patients who came to the outpatient and inpatient oncology department at UoGCSH. All cancer patients who visited the oncology department during the study were included. However, cancer patients who cannot communicate and have severe psychiatric problems were excluded from the study. The sample size for the study was determined using single population proportion formula considering a 95% confidence level, 5% margin of error, 50% expected proportion of diabetes, and 10% non-response rate. Then, the final sample size was found to be 423. The sample was selected using a systematic random sampling technique with a skip interval of two.

### Variables and data collection procedure

The outcome variable for this study was elevated blood sugar level, which is defined as a patient who is in a prediabetic or diabetic state and it was extracted from the patient’s medical file. The blood sugar of every cancer patient was measured using a CareSenseN glucometer. DM was diagnosed based on WHO and American Diabetes Association(ADA) guidelines which is, fasting blood sugar (FBS) ≥ 126 mg/dl or 2-h plasma glucose(PG) during Oral Glucose Tolerance Test(OGTT) ≥ 200 mg/dl^23,24^ or self-report of previous diagnosis of DM by health professional or currently taking treatment for diabetes. Besides, prediabetes was defined as a patient with FBS 100–125 mg/dl or 2-h PG during OGTT 140–199 mg/dl^23,24^. Socio-demographic characteristics of the patients, such as age, sex, residence, religion, occupation, and income, were assessed. In addition, behavioral characteristics such as cigarette smoking, alcohol consumption, Khat chewing, physical activity, and type of oil were assessed. Furthermore, cancer-related characteristics such as stage of cancer, duration of cancer, duration of treatment, treatment type, and metastasis were extracted from patient’s medical file. Physical measurements such as weight, height, waist circumference (WC) and blood pressure (BP) were measured. BP was measured three times in a sitting position using standard mercury sphygmomanometer. BP cuff with the appropriate cuff size that covers two-thirds of the upper arm was used. The measurement was taken after the participant rested for at least five minutes and assuring no smoking or caffeine 30 min before measurement. The second and the third measurements were taken five-to-ten minutes after the first and the second measurement, respectively. Finally, the average of the three BP measurements was calculated to determine the BP status of the participant. An individual was diagnosed as hypertensive if the systolic blood pressure (SBP) is ≥ 140 mg/dl or the diastolic blood pressure (DBP) is ≥ 90 mg/dl, or previous diagnosis of hypertension or current use of anti-hypertensive drug^25^. WC was measured at an approximate mid-point between the lower margin of the lowest palpable rib and the top of the iliac crest using flexible plastic tape without heavy outdoor closing. WC of ≥ 94 for males and ≥ 80 for females was considered high risk^26^. Weight and height to calculate BMI and were measured using calibrated equipment and BMI was calculated by dividing weight in kg by height in meters square. BMI < 18.5 kg/m^2^ was considered underweight, 18.5–24.9 kg/m^2^ as normal, 25–29.9 kg/m^2^ as overweight, and ≥ 30 kg/m^2^ as obese^27^.

A structured interviewer-administered questionnaire was used for data collection, and it was collected by four general practitioners working in the oncology department under the supervision of the principal investigator and one oncology specialist doctor. First, the patients were interviewed about their socio-demographic and behavioral characteristics. Then, all physical measurements were done, and finally, blood sugar levels and cancer-related characteristics were extracted from the medical file of patients. Both the interview and measures were completed at a single time point. The questionnaire was prepared in English, then translated to Amharic, then back-translated to English to maintain consistency. All the data collectors and supervisors took a one-day training on how to collect data. The principal investigator and the supervisor checked the collected data for its completeness and consistency daily.

### Data processing and analysis

The survey data were entered into Epidata version 3.1 and analyzed by STATA 14 software. Descriptive statistics were used to describe the study population in relation to different variables, and it is presented using texts, graphs, and tables. The chi-square assumption was checked for all categorical independent variables. A binary logistic regression model was used to identify factors associated with elevated blood sugar. Both bi-variable and multivariable logistic regression models were carried out. Variables with a p-value of less than 0.2 in the bi-variable analysis were entered into the multivariable analysis. Both Crude Odds Ratio (COR) and AOR with a 95% confidence interval were estimated to show the strength of associations. Finally, p-value < 0.05 in the multivariable logistic regression analysis was used to declare a statistically significant association. Hosmer and Lemeshow goodness of fit test was used to check the goodness of fit of the model. All methods were performed in accordance with the relevant guidelines and regulations.

### Ethics approval and consent to participate

The study protocol was approved by the ethical review committee of the college of Medicine and Health Sciences, University of Gondar. A letter of permission was also obtained from the oncology department. Informed consent was obtained from all subjects and/or their legal guardian. Respondents’ names and other personal identifiers were not included to keep confidentiality. The collected data was password protected.

## Results

### Socio-demographic and behavioral characteristics

A total of 384 cancer patients were included in the analysis after a response rate of 90.8%. More than half, 197(51.3%) of the participants were female, and 215(55.1%) were in the age group 41–60 years. More than two-thirds, 246(64.4%) of the participants were from rural areas, and 42(17%) had diabetes. Of the patients, 144(37.5%) were farmers and 87(60.4%) had prediabetes. Regarding alcohol consumption, 179(46.6%) are current alcohol consumers, and most 115(64.2%), have prediabetes. More than half, 202 (52.6%) of the participants had poor physical activity, and 172(45.6%) used liquid oil for cooking (Table 1).

**Table 1:** Socio-demographic and behavioral characteristics of cancer patients attending the oncology department at the University of Gondar comprehensive specialized hospital, 2021.

Variable	Normal (%) n = 102	Prediabetes (%) n = 218	Diabetes (%) n = 64	Total (%) n = 384	*P*-value
**Sex**					0.052
Male	40(39.22)	117(53.67)	30(46.88)	187 (48.70)	
Female	62(60.78)	101(46.33)	34(53.13)	197(51.30)	
**Age**					0.763
18–40	28(27.45)	55(25.23)	16(25.00)	99(25.78)	
41–60	57(55.88)	120(55.05)	38(59.38)	215(55.99)	
> 60	17(16.67)	43(19.72)	10(15.63)	70(18.23)	
**Residence**					0.848
Urban	38(37.62)	77(35.32)	21(33.33)	136(35.60)	
Rural	63(62.38)	141(64.68)	42(66.67)	246(64.40)	
**Religion**					0.032
Orthodox	55(53.92)	151(69.27)	37(58.81)	243(63.28)	
Muslim	30(29.41)	48(22.02)	13(20.31)	91(23.70)	
Protestant	15(14.71)	18(8.26)	12(18.75)	45(11.72)	
Others	2(1.96)	1(0.46)	2(3.13)	5(1.30)	
**Occupation**					0.949
Farmer	36(35.29)	87(39.91)	21(32.81)	144(37.50)	
Merchant	22(21.57)	45(20.64)	15(23.44)	82(21.35)	
Government employed	32(31.37)	66(30.28)	21(32.81)	119(30.99)	
Other	12(11.76)	20(9.17)	7(10.94)	39(10.16)	
**Income**					0.718
200–1200	32(31.37)	62(28.44)	18(28.13)	112(29.17)	
1201–4600	63(61.76)	145(66.51)	40(62.50)	248(64.58)	
> 4601	7(6.86)	11(5.05)	6(9.38)	24(6.25)	
**Cigarette smoking**					0.311
Never	91(89.22)	179(82.11)	52(81.25)	322(83.85)	
Used to smoke	1(1.96)	10(4.59)	5(7.81)	17(4.43)	
Current smoker	9(8.82)	29(13.30)	7(10.94)	45(11.72)	
**Alcohol consumption**					0.042
Never	61(59.80)	91(41.74)	31(48.44)	183(47.66)	
Used to drink	6(5.88)	12(5.50)	4(6.25)	22(5.73)	
Current drinker	35(34.31)	115(52.75)	29(45.31)	179(46.61)	
**Khat chewing**					0.546
Yes	24(23.53)	59(27.06)	20(31.25)	103(26.82)	
No	78(76.47)	159(72.94)	44(68.75)	281(73.18)	
**Physical activity**					0.029
Poor	5(4.90)	32(14.68)	13(20.31)	202(52.60)	
Moderate	37(36.27)	71(32.57)	24(37.50)	132(34.38)	
Vigorous	60(58.82)	115(52.75)	27(42.19)	50(13.02)	
**Oil type**					0.504
Cruddy oil	36(35.64)	69(32.55)	18(28.13)	123(32.63)	
Liquid oil	39(38.61)	101(47.64)	32(50%)	172(45.62)	
Butter	26(25.74)	42(19.81)	14(21.88)	82(21.75)	

### Cancer-related and clinical characteristics

Of the total participants, the majority, 233(58.4%), were in stage I cancer and 264(68.8%) had no metastasis. Regarding cancer treatment, 317(82.6) have already started treatment; of these, more than one-third, 123(38.8%), had surgery. The majority, 178(46.4%) of the cancer patients, knew their diagnosis before 3–7 months, and 28(15.7%) of them were diagnosed to have diabetes. Besides, 230(59.9%) of participants have cancer pain. Regarding BMI, only 47(12.2%) were underweight **(**Table 2**).**

**Table 2:** Cancer-related and other clinical characteristics of cancer patients attending the oncology department at the University of Gondar comprehensive specialized hospital, 2021.

Variable	Normal (%) n = 102	Prediabetes (%) n = 218	Diabetes (%) n = 64	Total (%) n = 384	*P*-value
**Stage of cancer**					0.989
Stage I	63(61.76)	125(57.87)	35(54.69)	233(58.38)	
Stage II	17(16.67)	40(18.52)	13(20.31)	70(18.32)	
Stage III	13(12.75)	30(18.52)	9(14.06)	52(13.61)	
Stage IV	9(8.82)	21(9.72)	7(10.94)	37(9.69)	
**Metastasis**					0.769
Yes	29(28.43)	70(32.11)	21(32.81)	120(31.25)	
No	73(71.57)	148(67.89)	43(67.19)	264(68.75)	
**Treatment for cancer**					0.593
Yes	85(83.33)	182(83.49)	50(70.13)	317(82.55)	
No	17(16.67)	36(16.51)	14(21.88)	67(17.45)	
**Treatment type (n = 317)**					0.422
Chemotherapy	20(23.53)	52(28.57)	8(16.00)	80(25.24)	
Surgery	34(40.00)	69(37.91)	20(40.00)	123(38.80)	
Surgery & Chemotherapy	31(36.47)	61(33.52)	22(44.00)	114(35.96)	
**Duration of cancer treatment (n = 317)**					0.834
≤ 2	26(30.23)	47(28.52)	13(26.53)	86(27.13)	
3–6	39(45.35)	95(52.20)	23(46.94)	157(49.53)	
≥ 7	21(24.42)	40(21.98)	13(26.53)	74(23.34)	
**Duration of cancer (in months)**					0.229
≤ 2	29(28.43)	72(33.03)	21(32.81)	122(31.77)	
3–7	43(42.16)	107(49.08)	28(43.75)	178(46.35)	
≥ 8	30(29.41)	39(17.89)	15(23.44)	84(21.88)	
**Cancer pain**					0.061
Yes	53(51.96)	132(60.55)	45(70.31)	230(59.90)	
No	49(48.04)	86(39.45)	19(29.69)	154(40.10)	
**Family history of DM**					NA
Yes	0(0.00)	0(0.00)	34(53.13)	34(8.85)	
No	102(100.00)	218(100.00)	30(46.88)	350(91.15)	
**Body Mass Index**					0.765
Underweight	12(11.76)	27(12.39)	8(12.50)	47(12.24)	
Normal weight	81(79.41)	179(82.11)	50(78.13)	310(80.73)	
Overweight	9(8.82)	12(5.50)	6(9.38)	27(7.03)	
**Central obesity**					0.059
Yes	26(25.49)	33(15.14)	15(23.44)	74(19.27)	
No	76(74.51)	185(84.86)	49(76.56)	310(80.73)	
**Hypertension**					0.008
Yes	69(67.65)	146(66.97)	30(46.88)	139(36.20)	
No	33(32.35)	72(33.03)	34(53.13)	245(63.80)	

NA: The variable doesn’t fulfill the assumption, and p-values cannot be produced.

### Prevalence of diabetes among cancer patients

**Figure 1:**
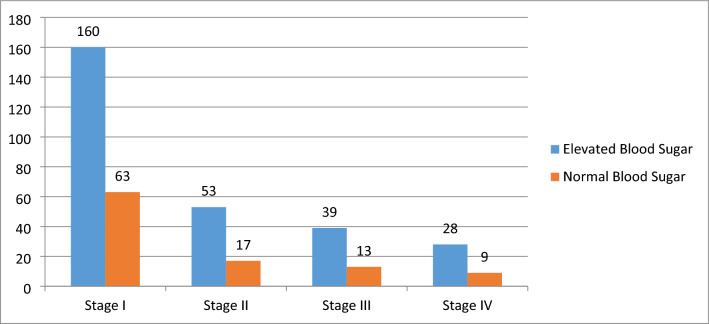
Multiple bar graph showing the frequency of elevated blood sugar across categories of cancer stage.

The proportion of elevated blood sugar among cancer patients was 73.4% (95% CI 68.8, 77.6). Of these, the proportion of prediabetes was 56.8% (95% CI 51.7, 61.7), and diabetes was 16.7% (95% CI 13.3, 20.8). Of the total DM cases identified, 26(40.6) were newly diagnosed, and the remaining were known DM patients on medication. The proportion of elevated blood sugar was highest (75.5%) among stage IV cancer patients and lowest (71.7%) among stage I (Fig. 1).

### Factors associated with elevated blood sugar level

Of the nine independent variables included in the multivariable model, only alcohol consumption was found to have a statistically significant association with elevated blood sugar among cancer patients. The odds of elevated blood sugar increased by 96% among cancer patients who consume alcohol compared to their counterparts keeping other variables constant (AOR: 1.96; 95%CI: 1.11, 3.46) (Table 3).

**Table 3:** Multivariable binary logistic regression table showing factors associated with elevated blood sugar among cancer patients attending the Oncology Department at the University of Gondar comprehensive specialized hospital, 2021.

Variable	Elevated blood sugar		COR (95% CI)	AOR (95% CI)	P-value
	Yes	No			
**Sex**					
Male	147	40	1	1	
Female	135	62	0.59(0.37, 0.94)	0.81(0.46, 1.43)	0.470
**Age in years**					
18–49	141	55	1	1	
≥ 50	141	47	1.17(0.74, 1.84)	1.16(0.69, 1.97)	0.577
**Residence**					
Urban	98	38	1	1	
Rural	183	63	1.12(0.70, 1.80)	1.31(0.75, 2.30)	0.342
**Alcohol consumption**					
No	122	61	1	1	
Yes	160	41	1.95(1.23, 3.09)	1.96(1.11, 3.46)	0.021
**Cigarette smoking**					
No	231	91	1	1	
Yes	51	11	1.82(0.91, 3.66)	0.82(0.35, 1.91)	0.641
**Stage of cancer**					
Stage I	160	63	1	1	
Stage II	53	17	1.23(0.66, 2.28)	1.37(0.66, 2.82)	0.394
Stage III	39	13	1.18(0.59, 2.36)	0.93(0.39, 2.25)	0.879
Stage IV	28	9	1.23(0.55, 2.74)	0.91(0.30, 2.72)	0.865
**Duration of cancer**					
≤ 4 months	178	55	1	1	
> 4 months	104	47	0.68(0.43, 1.08)	0.61(0.35, 1.05)	0.077
**Treatment type**					
Surgery	89	34	1	1	
Chemotherapy	60	20	1.15(0.60, 2.18)	0.94(0.46, 1.92)	0.856
Surgery & Chemo	83	31	1.02(0.58, 1.81)	0.82(0.40, 1.68)	0.585
**Cancer pain**					
No	105	49	1	1	
Yes	177	53	1.56(0.99, 2.46)	1.81(0.80, 3.77)	0.071

## Discussion

This study mainly investigated the prevalence of prediabetes and diabetes and its associated factors. The study found the proportion of diabetes and prediabetes among cancer patients to be 56.8% and 16.7%, respectively. Besides, alcohol consumption was found to be the independent risk factor for elevated blood sugar levels among cancer patients.

The proportion of prediabetes and diabetes in this study is higher than prospective cohort study conducted among cancer patients in the United States of America(USA), which reported the burden of prediabetes and diabetes at the end of the follow-up to be 21.2% and 32.6%, respectively^28^. The possible reason for this difference could be due to the kind of laboratory test used to assess the patient's blood sugar because the USA study uses hemoglobin A1c to diagnose prediabetes and diabetes in addition to FBS and RBS. Nonetheless, our study only uses FBS and OGTT to diagnose those diseases, which might increase our study's burden. The proportion of prediabetes and diabetes in this study is also much higher than other community-based studies conducted in Ethiopia which reported the burden of prediabetes and diabetes respectively to be 15.7% and 6.8%^29^, 9.3% and 6.3%^30^, 12% and 2.3%^31^, 15.9% and 6.5%^32^. This could be due to the difference in the study population since our study participants are only cancer patients, while the others are conducted in the general community. Both cancer and diabetes mellitus share common risk factors like age^6^. The risk of cancer increase with age due to the sequential accumulation of oncogenic mutations in a single clone and cell DNA damage accumulating over time^33^. Ageing also contributes to the pathogenesis of T2DM through the decreased β-cell function that accentuates the lack of insulin secretion^34^. In addition, cancer and DM share other risk factors such as obesity, physical inactivity, poor diet, alcohol consumption, and smoking^6^, which may increase the burden of elevated blood sugar among cancer patients. Insulin-like growth factor (IGF) is also a possible mechanism linking diabetes and cancer, as hyperinsulinemia causes a rise in the level of free and bioactive IGF-1, which increases the underlying risk of cancer in type 2 diabetic patients^35–37^. On the other hand, some studies suggest that cancer types such as pancreatic, colorectal and breast cancer increase the risk of diabetes^11,38–40^. Another reason for the higher prevalence in this study could be due to increased blood sugar caused by the cancer treatment. Corticosteroids are widely used for various purposes in patients with cancer, including for the prevention of chemotherapy-related hypersensitivity, management of brain metastasis^11^, and control of hematologic cancers^41^. These drugs are associated with an increased risk of hyperglycemia and diabetes since it causes reduced insulin sensitivity^12^, and this might increase the burden of diabetes among cancer patients. In addition, chemotherapies like L-asparaginase^42^, total body irradiation therapy^14^, and immunosuppressive agents such as Calcineurin inhibitors^13^ may also increase insulin resistance and the risk of elevated blood sugar. On the other hand, antidiabetic drugs such as pioglitazone, GLP-1 receptor antagonists, and insulin analogues are reported to be associated with bladder, medullary thyroid, and breast cancers, respectively, although there are some conflicting results^43^. Furthermore, cancer patients are more likely to lose weight and appetite, leading them to develop cancer cachexia syndrome^11^. This syndrome is linked to impaired glucose tolerance and diabetes due to various mechanisms^44–46^.

In this study, cancer patients who consume alcohol were found to have increased odds of having elevated blood sugar compared to their counterparts. This aligns with other studies conducted in Ethiopia^47^ and elsewhere^48–50^. This could be due to the effect of alcohol consumption on decreasing the function of pancreatic β-cells and increasing insulin resistance which may lead to hyperglycemia^50^. Alcohol beverages may also directly increase the blood sugar level^51,52^.

Although this study is the first in Ethiopia, it has some limitations. Due to the study design's cross-sectional nature, there is a problem of a chicken or egg dilemma. In addition, the study does not use Hemoglobin Alc to diagnose prediabetes and diabetes, which may overestimate the burden of those diseases. The study included all types of cancers, and the relation between each cancer type with blood glucose is not assessed. Besides, important factors such as the dietary habits of patients and paraneoplastic phenomena were not assessed. As a hospital-based study with a small sample size, it is impossible to generalize the findings to the general community.

## Conclusion

The burden of prediabetes and diabetes is alarmingly high among cancer patients. Alcohol consumption was found to increase the odds of having elevated blood sugar among cancer patients. Hence, it is essential to recognize cancer patients are at high risk of having elevated blood sugar and design strategies to prevent this disease. It is also good to link cancer and diabetic clinics to give coordinated care that can improve the treatment outcome of both diseases. It is also recommended to advise cancer patients to avoid alcohol consumption. It is also important to do further prospective cohort studies to assess the temporal relationship between cancer and diabetes.

## Data Availability

Data will be available from the corresponding author upon request.

## Abbreviations

AOR: Adjusted odds ratio
BMI: Body Mass Index
BP: Blood pressure
CI: Confidence interval
COR: Crude odds ratio
CSA: Central statistical agency
DBP: Diastolic blood pressure
DM: Diabetes mellitus
FBS: Fasting blood sugar
FMOH: Federal ministry of health
IDF: International diabetes federation
IGF: Insulin-like growth factor
IQR: Inter quartile range
NCD: Noncommunicable disease
RBS: Random blood sugar
SBP: Systolic blood pressure
UoGCSH: University of Gondar comprehensive specialized hospital
USA: United States of America
WC: Waist circumference
WHO: World Health Organization
